# Endemic Goitre in the Highland District of Perthshire, Scotland

**Published:** 1915-02

**Authors:** 


### ABSTRACTS

---

**Endemic Goitre in the Highland District of Perthshire, Scotland.** N. Douglas Mackay, Aberfeldy, "Caledonian Med. Jour.," January, 1915. The goitrous districts of Scotland are comparatively small. Mitchell in 1862 described that of Dumfries; McKenzie in 1894, that of Lanarkshire; St. Lager in 1867 that of Fife, Arran and Perthshire. The present article deals with the "Loch Tay" district, 56° 20′ to 56° 40′ N. Lat. and 3° 40′ to 4° 20′ W. Long., approximately 15 by 30 miles. This is a mountainous, well watered region, glaciated, the rocks being mainly igneous with considerable limestone, containing 53.33% calcium carbonate and 7.87% magnesium carbonate. Acharn has 12 cases in a hundred population; westward 15 cases among 12 cottages, 10 cases in three families. Similar approximate figures are given for various other localities. 38 cases in children in 32 schools are cited. The author mentions the general principles of determining epidemicity: proportion of cretins (Neither cretins nor deaf mutes being noted in this area); ratio of males to females, here about 1 to 2, which is high; tendency to the disease in new comers (not noted in summer visitors); size attained by the goitre (usually small or moderate, symmetric, parenchymatous involvement tending to become fibro-cystic.); incidence among children (not definitely stated but implied as being relatively high). The general conclusion is that the etiologic factor, whatever it may be, is of low virulence and gradual in its action.

